# Postpartum Pneumomediastinum: An Uncommon Cause for Chest Pain

**DOI:** 10.1155/2010/956142

**Published:** 2010-12-01

**Authors:** Vladimir Revicky, Paul Simpson, David Fraser

**Affiliations:** Department of Obstetrics and Gynaecology, Norfolk and Norwich University Hospital, Colney Lane, Norwich NR4 7UY, UK

## Abstract

This case report refers to a 32-year-old primiparous woman with a mild asthma, who had a normal vaginal delivery in a birthing pool and developed an acute postpartum chest pain due to pneumomediastinum and subcutaneous chest emphysema. After 72 hours of observation, she was discharged home without any residual symptoms.

## 1. Introduction

Postpartum pneumomediastinum in association with subcutaneous emphysema was described for the first time by Louis Hamman in 1939 [1]. This condition, also referred to as Hamman's syndrome, is rare, with only about 200 cases reported worldwide [2] and an estimated incidence of 1 in 100,000 vaginal deliveries [3]. Despite its frightening presentation, the condition is usually minimal and the course is self-limiting [2]. Subcutaneous emphysema in the neck has also been linked to vomiting during pregnancy [4]. Chest pain is the most common symptom of pneumomediastinum although the severity depends on the location and amount of air [1]. Additional symptoms may include dyspnoea, cough, and palpitations. A definitive diagnosis is made radiographically [2]. This case report demonstrates possible difficulties in diagnosing Hamman's syndrome and potential confusion with more serious conditions with higher morbidity and mortality, such as pulmonary embolism, amniotic fluid embolism, myocardial infarction, pneumothorax, and aortic dissection.

## 2. Case Report

A 32-year-old Caucasian primiparous low-risk woman had a normal vaginal delivery in the birthing pool. Antenatally, she was noted to have a mild asthma for which she used salbutamol inhaler on an irregular basis. She was nonsmoker, BMI 29, with no other medical or surgical history of note. She had spontaneous onset of labour at term, 24 hours after spontaneous rupture of membranes. Her first stage lasted twelve hours and second stage two hours. She used Entonox for pain control and had a spontaneous vaginal delivery of a healthy girl weighting 3.65 kg with an intact perineum. Three hours following her delivery, she complained of chest pain and a swollen neck. On examination, her blood pressure was 120/80 mmHg, pulse 95/minute, and O2 saturation 99% on the air. On auscultation, her chest was clear with bilateral air entry with no crepitus. However, palpation of her neck and chest revealed subcutaneous crepitation. A diagnosis of subcutaneous emphysema was established by the attending obstetric specialist registrar. The chest X-ray was ordered to rule out pneumothorax. The opinion of a medical team was sought as well. A chest radiograph showed pneumomediastinum and subcutaneous emphysema of the neck but no pneumothorax. A gastrografin swallow fluoroscopy study, arranged to exclude a tear in the oesophagus, was normal. Seventy, two hours later a repeat chest radiograph showed a resolving pneumomediastinum with reduced subcutaneous emphysema. The patient was asymptomatic and was discharged from the hospital.

## 3. Conclusion

The Valsalva manoeuvre can cause a rupture of marginal alveoli with air entering into the mediastinum. This has been clearly illustrated with radiopaque perfluorocarbon in a patient with asthma and spontaneous pneumomediastinum [5]. Further, alveolar rupture is associated with asthmatic crises. Although, the patient in this case report had asthma, this was only mild with sporadically used inhalers. Therefore, asthma as a sole culprit of the pneumomediastinum in this case is doubtful. Previously, it has been thought that nulliparity and a prolonged second stage of labour with vigorous pushing were features of a labour complicated by pneumomediastinum. However, Reeder reviewed 187 reported cases and confirmed that although most women were primiparous, they had a longer second stage of labour and normal fetal size [6]. In this case report, the patient was primiparous and had a regular length of the first and second stages of labour. She delivered in a birthing pool with average pushing effort according to a birth-attending midwife. Her chest pain developed three hours after delivery, when medical help was sought. Chest pain in a pregnancy or postnatal period may trigger a pathway of investigations for a pulmonary embolism, including CT scans or ventilation-perfusion lung scans with their radiation doses. The delivery of 10 mGy of radiation to a woman's breast increases her lifetime risk of developing breast cancer by 13.6% [7]. However, by meticulous clinical examination, including auscultation and palpation of chest, it should be possible to ascertain “crackles or bubbles sounds” with each heartbeat or to palpate crepitation subcutaneously. These classical physical findings, also known as Hamman's signs, were described in all cases of pneumomediastinum reported by Hamman [1]. There are no reports of the coexistence of pneumomediastinum with any of the embolic disorders and rarely with pneumothorax [3]. Thus, the only radiological modality verifying the diagnosis of pneumomediastinum and subcutaneous air is a plain chest radiograph, as carried out in this case. However, chest radiography may not be sufficient in demonstrating pneumomediastinum in some cases, therefore CT imaging may be necessary. Confirmation of air in the mediastinum usually precludes the need for additional evaluation to rule out pulmonary embolism, amniotic fluid embolism, myocardial infarction, and aortic dissection [3]. Contrast radiology is useful if there is a reason to suspect an oesophageal tear (Boerhaave's syndrome) [8].

Treatment of Hamman's syndrome is supportive. Chest pain, dyspnoea, and anxiety can be treated with oxygen, analgesics, and sedatives. Generally, Hamman's syndrome has a self-limiting course [2]. Following a comprehensive literature search, there are no reliable data on the rate of recurrence of this condition. Kobak and Abrams back in 1949 recommended the use of forceps routinely in any subsequent pregnancy [9]. Seidl and Brotzman (1994) described consequent spontaneous vaginal deliveries occurring without incident [2]. However, the management of subsequent deliveries remains contentious [10]. Entonox (nitrous oxide and oxygen) leads to a substantial expansion of trapped gases hence it should be avoided [11]. Maternal involuntary pushing may be effectively controlled with an epidural analgesia [11].
